# Structures and Role of the Intermediate Phases on the Crystallization of BaTiO_3_ from an Aqueous Synthesis Route

**DOI:** 10.1021/acsomega.1c00089

**Published:** 2021-03-30

**Authors:** Kristine Bakken, Viviann H. Pedersen, Anders B. Blichfeld, Inger-Emma Nylund, Satoshi Tominaka, Koji Ohara, Tor Grande, Mari-Ann Einarsrud

**Affiliations:** † Department of Materials Science and Engineering, NTNU Norwegian University of Science and Technology, Trondheim 7491, Norway; ‡ International Center for Materials Nanoarchitectonics, National Institute for Materials Science, Ibaraki 305-0044, Japan; § Diffraction and Scattering Division, Center for Synchrotron Radiation Research, Japan Synchrotron Radiation Research Institute, Hyogo 679-5198, Japan

## Abstract

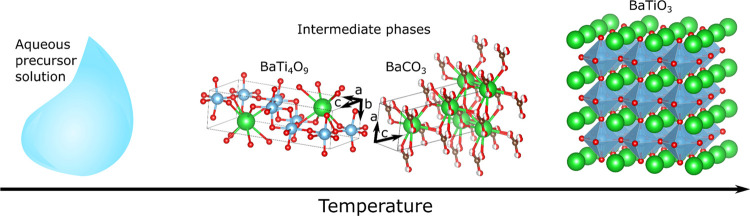

Carbonate formation is a prevailing challenge in synthesis of BaTiO_3_, especially through wet chemical synthesis routes. In this work, we report the phase evolution during thermal annealing of an aqueous BaTiO_3_ precursor solution, with a particular focus on the structures and role of intermediate phases forming prior to BaTiO_3_ nucleation. *In situ* infrared spectroscopy, *in situ* X-ray total scattering, and transmission electron microscopy were used to reveal the decomposition, pyrolysis, and crystallization reactions occurring during thermal processing. Our results show that the intermediate phases consist of nanosized calcite-like BaCO_3_ and BaTi_4_O_9_ phases and that the intimate mixing of these along with their metastability ensures complete decomposition to form BaTiO_3_ above 600 °C. We demonstrate that the stability of the intermediate phases is dependent on the processing atmosphere, where especially enhanced CO_2_ levels is detrimental for the formation of phase pure BaTiO_3_.

## 1 Introduction

The thermodynamic stability of BaCO_3_ poses a common synthesis challenge for producing BaTiO_3_, which is a ferroelectric material widely used in capacitors.^1^ The stability of BaCO_3_ relative to BaTiO_3_ increases with a high partial pressure of CO_2_ in the atmosphere and analogously with the activity (concentration) of CO_2_ in water.^2^ Moreover, the solubility of BaCO_3_ in water is limited,^3^ which combined with the thermodynamic stability makes carbonate secondary phases prevalent both in solid-state reactions and aqueous processing of BaTiO_3_ materials. The affinity for carbonate formation arises from the basicity of BaO combined with the abundance of CO_2_ in the atmosphere but also dissolved in water. Similar synthesis challenges are observed in other Ba-containing oxides or ceramics consisting of basic oxides.^4^

Wet chemical synthesis studies of BaTiO_3_-based powders and thin films generally report BaCO_3_ compounds as intermediate and secondary phases.^1,5^ Using sol–gel-related methods based on organic solvents, the precursors tend to decompose to form aragonite-type carbonate (BaCO_3_ (A)) and TiO_2_, where BaTiO_3_ nucleates through the solid-state reactions of these above 600 °C.^6−10^ However, in Pechini-based synthesis methods and from aqueous processing, a so-called oxycarbonate phase with the global proposed stoichiometry Ba_2_Ti_2_O_5_CO_3_ is observed.^10−20^ Recently, aqueous chemical solution deposition (CSD) synthesis routes for BaTiO_3_-based thin films have been reported,^21−23^ where this intermediate oxycarbonate phase was observed to form prior to the perovskite, dependent on the thermal processing. The pyrolysis reactions and the formation of the oxycarbonate were reported to play an integral part in the texture formation, phase purity, and quality of the films.^22,23^ The oxycarbonate seems to inhibit the formation of BaCO_3_ (A) and therefore also shifts the BaTiO_3_ formation from the solid**-**state reaction of TiO_2_ and BaCO_3_ (A) to proceed through decomposition of the oxycarbonate. The formation of the intermediate oxycarbonate phase has been linked to the presence of a “carbonate-like” linkage in the barium carboxylates^24^ or to the formation of a mixed metal citric acid complex in the precursor solution.^12−17^

Several local structures for the oxycarbonate phase have been proposed based on the overall global structure of Ba_2_Ti_2_O_5_CO_3_.^15−18^ However, Ischenko *et al.* observed that the intermediate oxycarbonate locally consisted of Ba- and Ti-rich nanosized regions, where there was no clear single crystalline structure in the Ti-rich area but instead a range of Ba*_y_*TiO_2+*y*_ phases were observed.^19^ The Ba-rich areas had a structure close to the high-temperature calcite-type polymorph of BaCO_3_ (*R*3̅*mH*, no. 166),^19,20^ where substitution of CO_3_^2–^ with O^2–^ stabilized the calcite structure giving BaO*_x_*(CO_3_)_1–*x*_.^19,20^ The proposed general reaction for the transformation pathway of BaTiO_3_ was through the formation of this intermediate oxycarbonate phase:
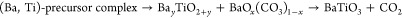
1

The BaO*_x_*(CO_3_)_1–*x*_ phase was observed to form preferably in the presence of titanium.^19,20^ Hence, a second stabilizing mechanism was also proposed by Ischenko *et al.*: topotaxial formation of structural domains of calcite-type carbonate (BaCO_3_ (C)) by templating with oxygen-deficient Ti-rich BaTiO_3_-like structures.^20^ A comparison of the aragonite and calcite modifications of BaCO_3_ can be found in Table 1. Although the models for the intermediate phases have been proposed, it is not known how these phases are affected by processing conditions, such as the heating rate, annealing temperature, and atmosphere, and the intermediate phases influence the formation of BaTiO_3_.

**Table 1:** Comparison of the Structures of the Aragonite- and Calcite-Type BaCO_3_^a^

	aragonite BaCO_3_	calcite BaCO_3_
crystal structure	*Pmcn* (nr. 62)	*R*3̅*mH* (nr. 160)
IR-active absorption bands [cm^–1^]	697, 856, 1059, 1435	693, 875, 1059, 1390–1435
bond lengths [Å]		
Ba–Ba	4.35, 4.357, 4.528	4.615
B–C	3.19, 3.247, 3.63, 3.734	3.35, 3.637, 4.954
Ba–O	2.705, 2.708, 2.783, 2.824, 2.86, 4.394, 4.398, 4.467, 4.486, 4.513, 4.556, 4.611, 4.647, 4.858	2.706, 3.004, 4.296, 4.49

a Based on refs^19, 20, 34, 36, 38^

*In situ* characterization is a rapidly growing field as studying materials under real-time conditions is necessary for further development, especially in battery research^25,26^ and hydrogen technology.^27^ Moreover, as new synthesis methods are implemented for complex material systems, much insight into the reaction pathway is required, where *in situ* techniques are becoming more prominent.^28−31^*In situ* studies by X-ray diffraction on BaTiO_3_ are reported both during deposition^32,33^ and the annealing^22^ of thin films.

Here, we report the thermal decomposition and phase evolution for BaTiO_3_ powders from an oxycarbonate forming aqueous synthesis route. *In situ* infrared (IR) spectroscopy and synchrotron X-ray total scattering were used to study the decomposition of the precursor, formation of intermediate phases, and nucleation of BaTiO_3_ from the intermediate phases. Moreover, the effect of partial pressure of CO_2_ during the decomposition was investigated. Rietveld and pair distribution function (PDF) refinements revealed the global and local structures of the intermediate phases present in the powders before BaTiO_3_ nucleation, supported by electron microscopy. The intermediate phases were found to consist of BaCO_3_ (C) and a BaTi_4_O_9_ phase; however, the formation of these depends heavily on the processing conditions, especially the heating rate, and partial pressure of CO_2_.

## 2 Results

### 2.1 *In Situ* Characterization during Thermal Annealing of BaTiO_3_ Precursor Powders

**Figure 1:**
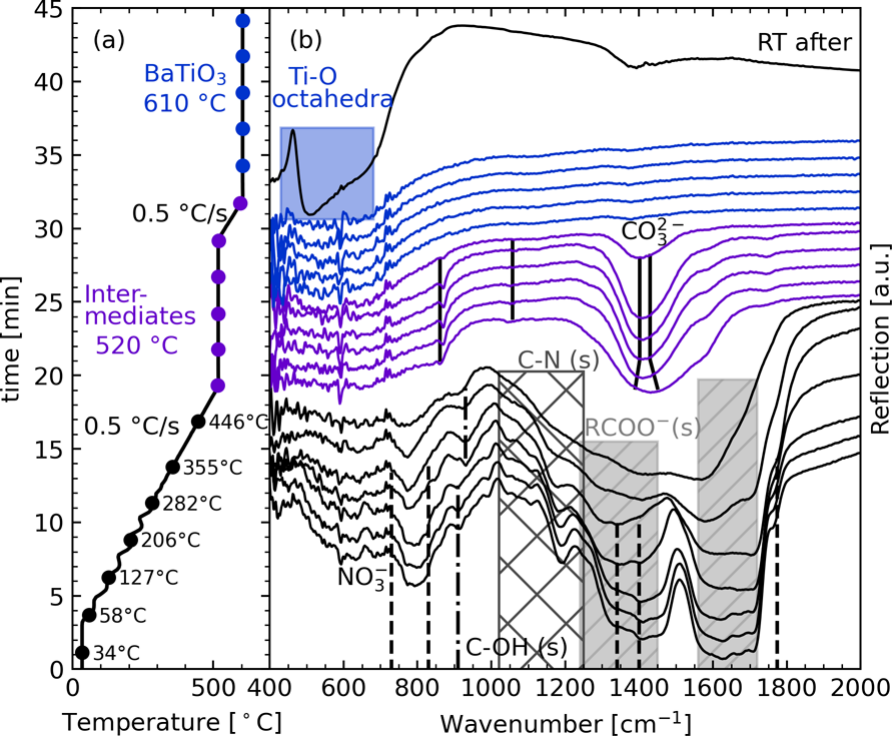
(a) Temperature profile and (b) *in situ* IR spectra of a BaTiO_3_ precursor powder showing the phase evolution during annealing in synthetic air to 610 °C with a hold step at 520 °C. Bands assigned to the functional groups in the precursor are indicated along with the BaCO_3_ bands developing at higher temperatures, and the signature of the Ti–O octahedra band is indicated for the spectrum recorded at room temperature (RT after).

Figure 1 shows the *in situ* IR spectra of the BaTiO_3_ precursor powder during annealing in synthetic air with a hold step at 520 °C to facilitate formation of the intermediate phases. (The full spectra are included in Figure S2.) The IR spectrum of the amorphous as-prepared precursor contained a split asymmetric stretching (as) mode (1340 and 1400 cm^–1^) of NO_3_^−^, showing a perturbation of the nitrate ion by cation interactions.^34,35^ The symmetric stretching (ss, 1240–1450 cm^–1^) and as- (1590–1750 cm^–1^) modes for the carboxylic acid groups originating from EDTA, citric acid, and their derivatives are indicated as wide bands, reflecting bonding to different metal ions.^34,35^ The frequency range for the as-band of the nitrate and carboxylic acid groups overlap, so the intensity of the wide band also has a contribution from the nitrate. The characteristic C–OH out-of-plane (oop) bending mode for carboxylic acid groups was observed at 930 cm^–1^. The C–N stretching mode (1020–1250 cm^–1^) from the EDTA derivatives was identified as a wide band.^34,35^ As the temperature is increased above 200 °C, the intensity of the bands decreases due to the decomposition of the nitrate. Between 300 and 400 °C, the bands assigned to the different functional groups in the precursor merge to a wide band (1000–1800 cm^–1^). In the temperature range of 470–520 °C, this wide band narrows to the asymmetric stretching band of the carbonate ion. Carbonate species remain in the powder until 610 °C where there is only BaTiO_3_, as seen from the absence of other bands in the *in situ* spectra and from the *ex situ* spectrum taken from the same sample after cooling (marked “RT after”). These observations correspond well with the IR spectra reported for calcined powders from the same precursor solution, where the carbonate absorption band was observed in the temperature range of 450–650 °C.^21^

**Figure 2:**
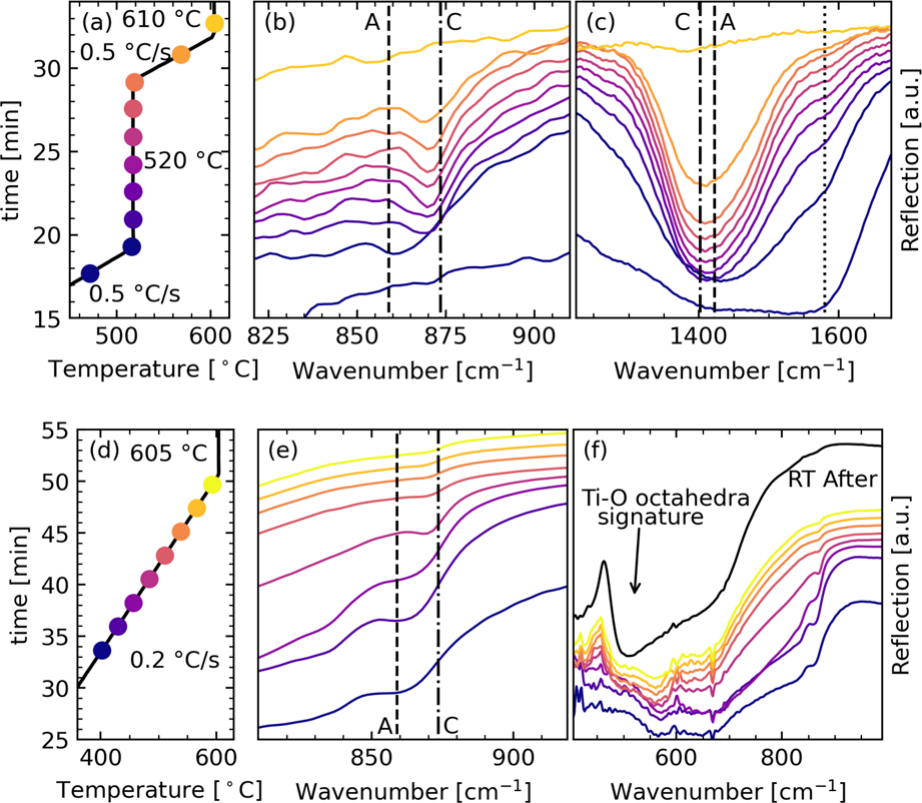
*In situ* IR spectra of BaTiO_3_ precursors annealed with different temperature programs. (a) Temperature profile and IR spectra in the frequency range of (b) the out-of-plane vibrational mode and (c) the asymmetric stretching mode of BaCO_3_, measured in synthetic air. (d) Temperature profile and IR spectra in (e) the frequency range for the out-of-plane vibrational mode of BaCO_3_ and (f) the low frequency range, measured in an ambient atmosphere and without instrument vacuum. The absorption bands of BaCO_3_ (A) are indicated as A and of BaCO_3_ (C) as C.

The IR spectra from Figure 1 in the temperature region where carbonate species are present are shown in greater detail in Figure 2a–c. A shift in the carbonate frequencies was observed for both the oop and as-bands with increasing temperature from those of BaCO_3_ (A) (861 and 1430 cm^–1^) to those of BaCO_3_ (C) (871 and 1402 cm^–1^),^34^ which is a commonly reported feature of the intermediate oxycarbonate phase.^15−20^ Broad bands developed at the start of the hold period became sharper with prolonged annealing, and the frequencies shifted toward that of calcite. Isothermal formation of the intermediate phases and heating with a low heating rate (Figure S3) were also investigated and showed the same trends for the carbonate band development. The wide carbonate as-band was also accompanied by shoulders at 1580 and at 1280 cm^–1^ in some cases (summarized in Table S1). It is proposed that the shoulders could be assigned to a splitting of the asymmetric stretching band of CO_3_^2–^ bonded to Ti^4+^ as the presence of titanium is necessary for the BaCO_3_ (C) formation (Figure S4).

To ease the comparison of the development of the bands assigned to the perovskite and the carbonate, a BaTiO_3_ precursor powder was heated without the dome of the reaction chamber and no instrument vacuum. The *in situ* IR spectra of the precursor in the temperature region of the formation of intermediates and decomposition are shown in Figure 2d–f. The carbonate oop-band (Figure 2e) and as-band (not shown) were visible above 400 °C, similar to the sample with a hold step (Figure 2b). The oop-band was seen as a wide feature at the aragonite frequency (859 cm^–1^) but shifted toward that of calcite (871 cm^–1^) as the temperature increased. No shoulder accompanying the as-band was observed for this heating program. The signature of the perovskite band associated with the Ti–O octahedra (Figure 2f) was observed at 500 °C and became more pronounced as the temperature increased. The formation of the perovskite band (Figure 2f) was accompanied with the formation of BaCO_3_ (C) (Figure 2e), and as the perovskite band became more pronounced, the carbonate oop-band decreased in intensity, showing how the BaCO_3_ (C) decomposes and BaTiO_3_ is formed.

**Figure 3:**
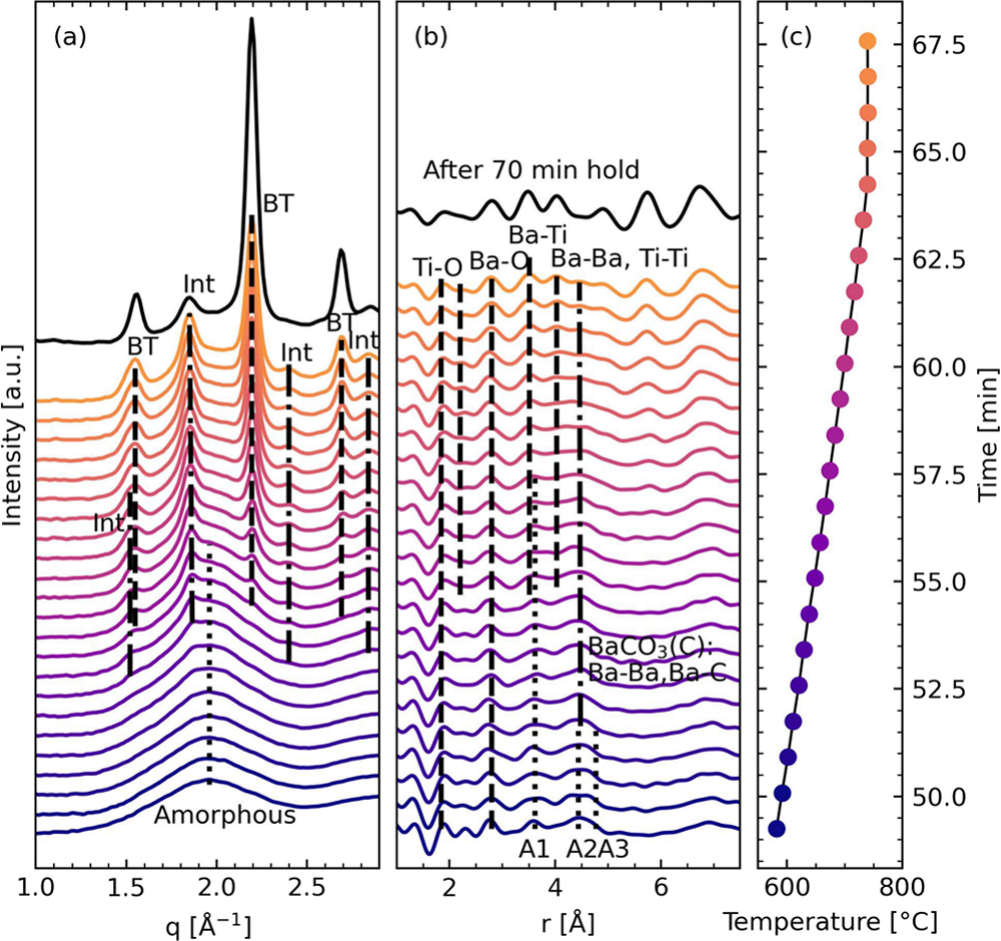
(a) Measured total scattering, (b) the converted PDFs, and (c) temperature profile for a BaTiO_3_ precursor powder heated with a heating rate of 0.17 °C/s to 734 °C in synthetic air. The diffraction lines in panel (a) are indicated as “BT” for BaTiO_3_ and “Int” for the intermediate BaCO_3_ (C) and BaTi_4_O_9_ phases. A broad feature corresponding to amorphous aragonite-like carbonate is also indicated. The atomic pair distances are indicated in panel (b) for the BaTiO_3_ phase, BaCO_3_ (C), and an amorphous phase. In the amorphous phase, several overlapping atomic pair distances exist for each of the indicated peaks. Peak A1 corresponds to a Ba–C distance in BaCO_3_ (A) and Ba–Ba, Ba–Ti, and Ti–O distances in a BaTi_4_O_9_ phase. Peak A2 corresponds to Ba–Ba and Ba–O distances in BaCO_3_ (A). The A3 peak corresponds to a Ba–O distance in BaCO_3_ (A) and a Ba–Ti distance in the BaTi_4_O_9_ phase.

The total scattering patterns and converted PDFs of the BaTiO_3_ precursor powder heated *in situ* with a heating rate of 0.17 °C/s are shown in Figure 3 (and Figure S5). The diffraction patterns (Figure 3a) demonstrate that the sample is amorphous until diffraction lines corresponding to intermediate phases appear at 630 °C, quickly followed by BaTiO_3_ crystallization at 690 °C. The crystallinity of the BaTiO_3_ phase is poor but increases during a hold period of 70 min at 734 °C. The PDF (Figure 3b) further demonstrates that the sample is amorphous at low temperatures but transforms into the intermediate phases with increasing temperature and that no long-range order is observed until crystallization of BaTiO_3_ occurs. The shortest Ti–O and Ba–O bonds (marked in Figure 3) remain unchanged even going from the amorphous phases at low and intermediate temperatures and through crystallization of BaTiO_3_. Three additional peaks were identified in the PDFs of the amorphous powder, and a certain local structural change is seen upon formation of the intermediate phases. No long-range structure is observed, indicating that the intermediate phases are nanocrystalline. BaTiO_3_ crystallization was accompanied by the emergence of peaks in the PDFs from the periodic Ba–Ti, Ba–Ba, and Ti–Ti distances and progressively enhanced long-range order.

### 2.2 Structural Investigation of the Intermediate Phases

**Figure 4:**
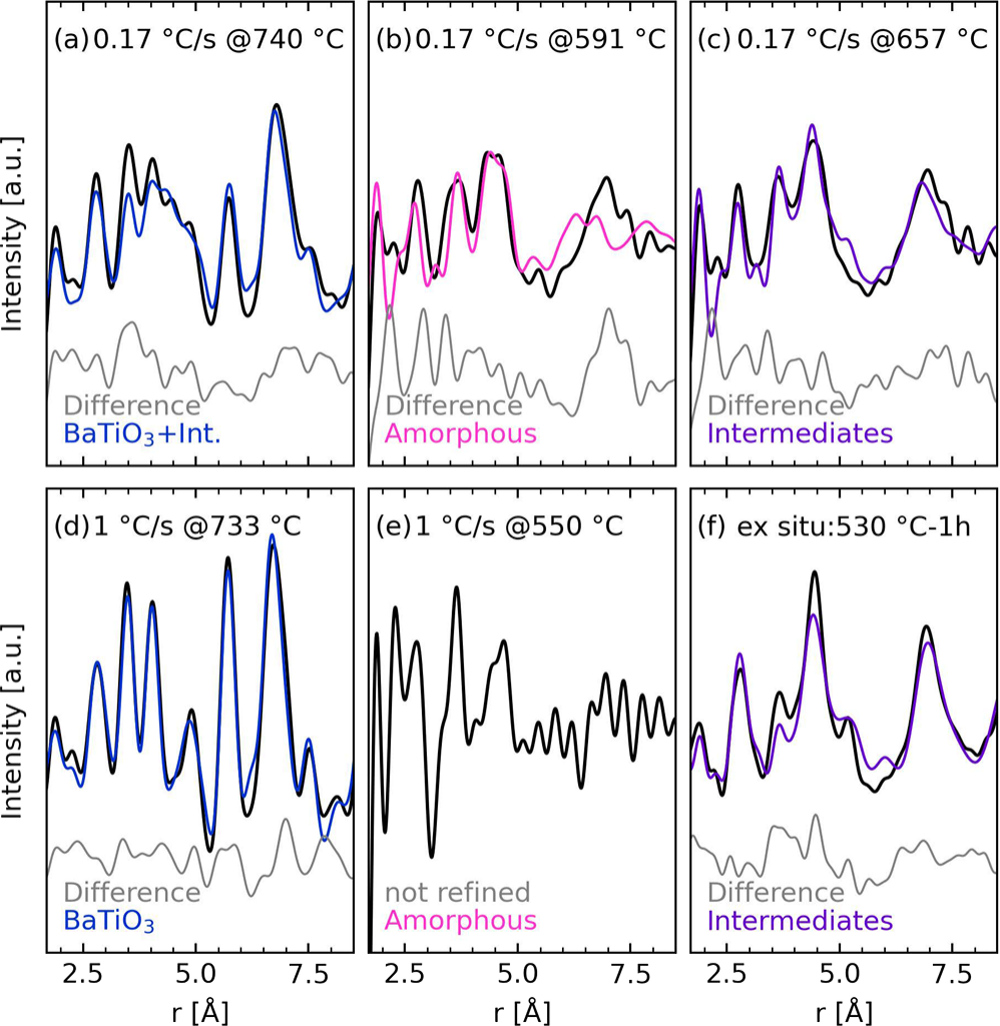
Converted and refined PDFs for BaTiO_3_ precursor powder with different annealing conditions. (a–c) BaTiO3 precursor powder measured during *in situ* heating with a 0.17 °C/s heating rate in air. (d,e) BaTiO_3_ precursor powder measured during *in situ* heating with a 1 °C/s heating rate in air. (f) BaTiO_3_ precursor powder annealed *ex situ* at 530 °C for 1 h in air, total scattering data recorded at ambient temperature. Intermediate phases are marked “Int” or “intermediates”.

Selected converted and refined PDFs are shown in Figure 4 for BaTiO_3_ precursor powders during different stages of the thermal annealing. The refinement of crystalline BaTiO_3_ (Figure 4a,d) shows a reasonable fit using a rhombohedral local structure for BaTiO_3_ (*R*3*m*, nr. 160^36−38^), although there are deviations indicating a form of local disorder that could not be refined with periodic structures. The refined PDF for the crystallized powder heated with 0.17 °C/s (Figure 4a) also has a contribution from the intermediate phases. The refined PDF for the amorphous powder (prior to any crystallization) is shown in Figure 4b (0.17 °C/s), where the nearest atomic pair distances found in the BaCO_3_ (A) and BaTi_4_O_9_ structures correspond well with the experimental data, but these phases do not give the correct long-range structure. Using 1 °C/s, the PDFs of the amorphous powder (Figure 4e, the full temperature range is shown in Figures S6 and S7) show sharp and narrow peaks. Both amorphous powders (Figure 4b,e) have peaks that can be described by short-range distances found in BaCO_3_ (A) and BaTi_4_O_9_. The intermediate phases only formed using 0.17 °C/s (Figure 4c), which had a contribution to the PDF from the amorphous phase compared to the *ex situ* annealed powder (Figure 4f), impacting the scale factor and crystallite sizes. However, both PDFs were fitted with a BaCO_3_ (C) structure (*R*3*m*, nr. 160^38,39^) and a BaTi_4_O_9_ phase (*Pmmn*, nr. 59^37^), and the ratio between these was locked to 75:25 during refinements, keeping with the global stoichiometry. The CO_3_^2−^ in the calcite structure was allowed full rotational freedom. However, the carbonate groups were observed to only exhibit slight vibrations around their equilibrium position, which eliminate the mirror plane compared to the calcite structure reported by Ischenko *et al*.^19,20^ There is an uncertainty associated with the refined structure and composition of the BaTi_4_O_9_ phase as the PDF pattern of the intermediates is largely dominated by the signal from the carbonate (Figure S8). Furthermore, the obtained fit of the intermediate phases indicates that a periodic structure cannot fully represent the local structure; hence, local disorder is expected. Refined lattice parameters for the PDFs displayed in Figure 4 are listed in Table S2.

**Figure 5:**
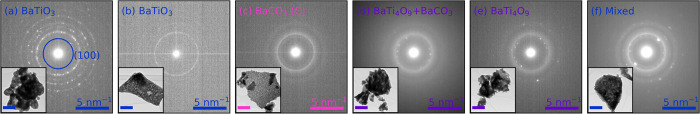
TEM SADP from the center of different particles in a BaTiO_3_ precursor powder annealed at 530 °C for 1 h in air. The insets show BF images of the particles, where the scale bars correspond to 200 nm. The imaged particles were identified as (a) BaTiO_3_, (b) BaTiO_3_, (c) BaCO_3_ (C), (d) BaTi_4_O_9_ and BaCO_3_ (A), (e) BaTi_4_O_9_, and (f) mixture of different phases.

The intermediate phases were also investigated by TEM imaging of a BaTiO_3_ precursor powder that was preannealed at 530 °C for 1 h. The TEM bright-field (BF) images and selected area diffraction patterns (SADPs) of selected particles are shown in Figure 5, demonstrating that the crystallinity of the intermediate phases was limited. However, both nanocrystalline and poorly crystalline BaTiO_3_ particles (Figure 5a,b, respectively) were observed in the powder even if the XRD patterns^21^ do not show BaTiO_3_ at this temperature (530 °C). Separate BaCO_3_ (C) (Figure 5c) and BaTi_4_O_9_ (Figure 5d,e) crystallites were also identified based on the SADP, although the crystallinity of these particles was also low. The diffraction from the presence of BaTi_4_O_9_ in Figure 5d also includes a contribution from BaCO_3_ (A) and possibly also BaCO_3_ (C). The particle in Figure 5f showed diffraction both from the BaCO_3_ (C) and BaTi_4_O_9_ phases. A large crystallite of the BaTi_4_O_9_ phase was probed, giving strongly directional diffraction (2 strong spots) with a *d*-spacing of 3.638 Å. This *d*-spacing, corresponding to the (110) planes, is slightly larger in the mixed particle (Figure 5f) than the diffraction spots seen in the BaTi_4_O_9_ particle (Figure 5e), illustrating the change in unit cell with increasing crystallinity. A weak diffraction ring with a *d*-spacing of 4.065 Å is observed in the particle in Figure 5f, which could correspond to the (101) diffraction line of BaCO_3_ (C), but it could also be the emerging (100) diffraction of BaTiO_3_. The identified *d*-spacings of the particles in Figure 5 are summarized in Table S3.

### 2.3 Variable CO_2_ Partial Pressure during Annealing of BaTiO_3_ Precursors

**Figure 6:**
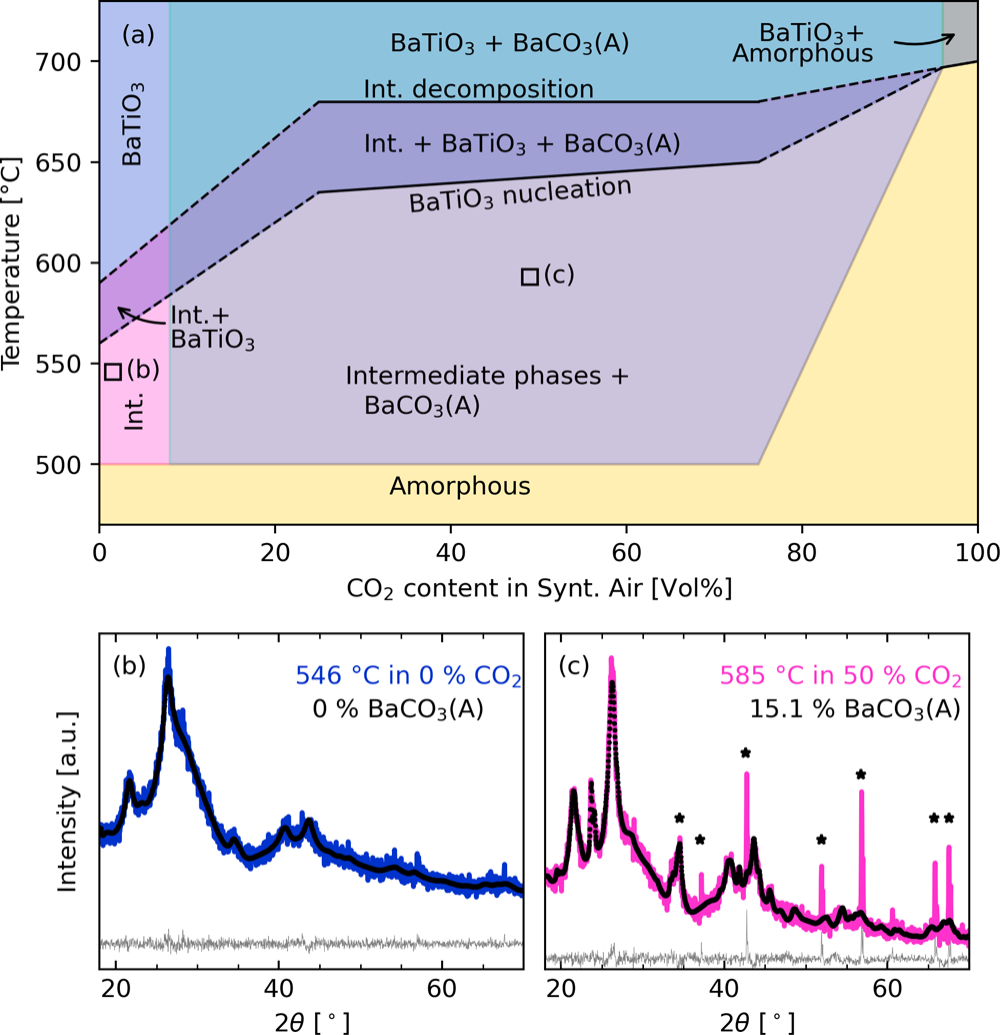
(a) Summary of the phases present as a function of both temperature and CO_2_ partial pressure during *in situ* HT-XRD measurements of BaTiO_3_ precursor powders. 0, 25, 50, 75, and 100 vol % CO_2_ were used in the experiments. Intermediate phases are marked “Int.”, and stippled lines are extrapolated boundaries. XRD patterns and Rietveld refinements of (b) BaTiO_3_ precursor powder heated *in situ* to 546 °C in synthetic air (0% CO_2_) and (c) BaTiO_3_ precursor powder heated *in situ* to 585 °C in 50 vol % CO_2_. Sharp peaks from the alumina crucible are marked with asterisks.

Since CO_2_ is released during the decomposition and formation of BaTiO_3_ according to the proposed reaction in eq 1, the partial pressure of CO_2_ is an important processing parameter. The phases present in each recorded diffractogram during *in situ* HT-XRD as a function of temperature and CO_2_ partial pressure are summarized in Figure 6 (all measured XRD patterns are shown in Figure S9). The results for the 0% CO_2_ (synthetic air) correspond well with previous *ex situ* powder XRD results^21^ and *in situ* XRD of BaTiO_3_ films from the same precursor solution.^22^ The formation of the intermediate phases was unaffected by increasing CO_2_ partial pressure as they formed above 505 °C in all the gas mixtures, except in pure CO_2_. However, for the powders heated in a CO_2_-rich atmosphere, BaCO_3_ (A) formed as a thermodynamically stable phase alongside the intermediates. The temperatures used during the *in situ* powder HT-XRD measurements were not sufficient to remove BaCO_3_ (A), so once formed the aragonite remained, even after formation of BaTiO_3_. However, due to stoichiometric concerns, there should also be Ti-rich phases present, but since these are amorphous, they do not appear in the XRD patterns. The diffractogram of the sample heated to 546 °C in synthetic air (Figure 6b) demonstrates the presence of only the intermediate phases (BaCO_3_ (C) and BaTi_4_O_9_), while at 585 °C in 50% CO_2_ (Figure 6c), the powder contains about 15 wt % BaCO_3_ (A) in addition to the intermediate phases. The refined cell parameters and crystallite sizes from the Rietveld refinements are listed in Table S4, where the poor crystallinity in this temperature region resulted in small refined crystallite sizes for both BaCO_3_ (C) and BaTi_4_O_9_.

The stability of intermediate phases over BaTiO_3_ nucleation exhibited a strong CO_2_ dependence even though the formation temperature for the intermediates was unaffected by the CO_2_ partial pressure. In synthetic air, the intermediates decomposed below 600 °C, while in a CO_2_-rich atmosphere, they were still present at 675 °C (Figure 6a), which likely is caused by a stabilization of the BaCO_3_ (C) phase by CO_2_. The increased carbonate stability also affected the BaTiO_3_ nucleation temperature, which was below 565 °C in synthetic air, but increased to above 650 °C in the CO_2_-rich atmosphere. There were no significant differences in the phase evolution of the samples heated in 25–75% CO_2_.

## 3 Discussion

### 3.1 Transformation Pathway: Decomposition and Pyrolysis

**Figure 7:**
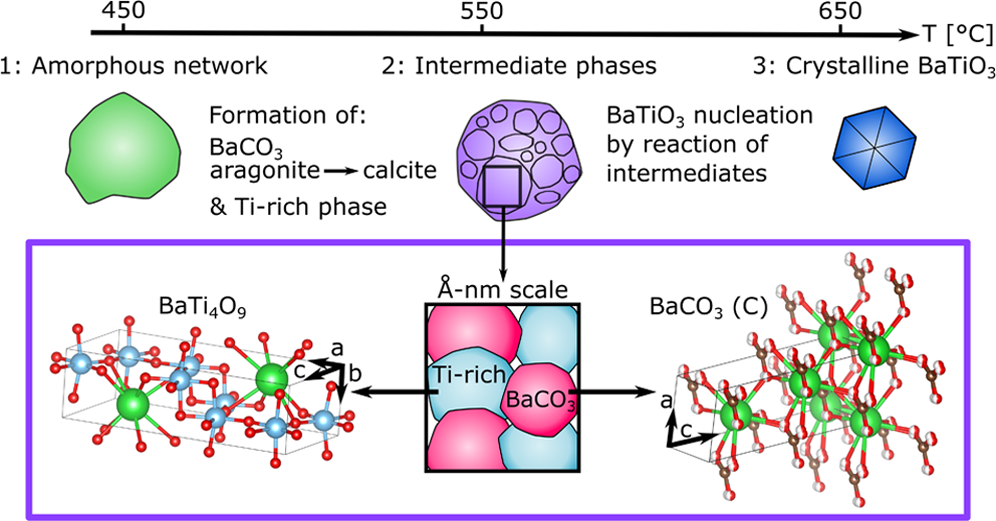
Illustration of the transformation pathway for BaTiO_3_ precursor powders from an aqueous solution, from an amorphous network (step 1) at low temperatures to crystallization of BaTiO_3_ (step 3) through reaction of the intermediate phases (step 2). The intermediate phases consist locally of small domains of BaCO_3_ with a calcite structure and a Ti-rich phase with a BaTi_4_O_9_ structure, although there is limited long-range order for both phases. Structures made by VESTA.^40^

The basicity of BaO and high stability of BaCO_3_ make carbonate formation almost inevitable during the thermal processing of the BaTiO_3_ precursor powder from an aqueous synthesis route. However, the phase evolution of the powders studied in this work followed the “oxycarbonate forming route” commonly reported for Pechini and sol–gel-based synthesis, instead of the solid-state reaction route. Therefore, it is probable that a mixed metal citric acid complex similar to those reported in refs (12, 14) formed in the precursor solution. A proposed transformation pathway is illustrated in Figure 7, and the decomposition and pyrolysis reactions can be divided into the following steps:

#### 3.1.1 Decomposition (200–560 °C)

At the start of this period, the nitrate decomposes, ammonia evaporates, and the decomposition/combustion of the organics initiates (step 1 in Figure 7). However, the wide RCOO^–^ stretching bands are still present, as seen from the *in situ* IR spectra of the powder precursors (Figure 1), originating from a variety of organic groups in the sample. The symmetric and asymmetric stretching bands of the RCOO^–^ groups become narrower and shift toward the asymmetric carbonate band as temperature is increased, demonstrating the preference for carbonate formation in this system. The organic removal (pyrolysis) can be expressed through the following proposed reaction (adapted from Ischenko *et al*.^19^)
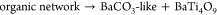
2which occurs at the initiation of this temperature region, leaving only BaCO_3_-like and BaTi_4_O_9_ phases in the system, with no long-range order. This is in accordance with the thermal analysis of the precursor powder, where a decrease in the mass fraction over a narrow temperature range is reported at 550 °C^21^ corresponding to removal of the remaining organic compounds.

#### 3.1.2 Carbonate Formation (480–570 °C)

In this temperature range, the IR spectra only showed bands corresponding to carbonate (Figures 1 and 2), which were initially broad features of an amorphous carbonate species with a frequency corresponding to BaCO_3_ (A) but quickly shifted toward the calcite frequency as the temperature was increased, according to the following proposed reaction
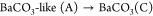
3

The carbonate frequency shift is accompanied by formation of a Ti-carbonate-like phase as indicated in the IR spectra (Figures 1 and 2), and weak reflections corresponding to the intermediate BaCO_3_ (C) and BaTi_4_O_9_ phases are observed in the XRD patterns (Figure 3). The refined structures of BaCO_3_ (C) and BaTi_4_O_9_ are displayed in Figure 7. Both the Rietveld and PDF refinements demonstrate that the crystallite size of each of the intermediate phases is in the nanorange or that the crystallinity is poor. This is further supported by the TEM images and SADP (Figure 5), showing limited crystallinity of the particles and small crystallites.

#### 3.1.3 BaTiO_3_ Nucleation and Growth (550–650 °C)

Nucleation of BaTiO_3_ occurs both directly from the amorphous phase during decomposition and through reaction of the intermediate phases according to the following proposed reaction (adapted from Ischenko *et al*.^19^)
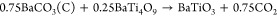
4

The nucleation of BaTiO_3_ occurs homogeneously throughout the powder as TEM showed that the carbonate and Ti-rich phases are intimately mixed, and the metastable nature of the intermediate phases ensures complete decomposition.

### 3.2 Structures of the Intermediate Phases

The intermediate phases were found to consist locally of poorly crystalline and nanosized BaCO_3_ (C) and BaTi_4_O_9_ with limited long-range order. Since BaCO_3_ (A) is the thermodynamically stable structure in the temperature range of carbonate formation (eq 3),^38^ the formation of BaCO_3_ (C) is likely enabled due to one or several stabilizing mechanisms. The presence of titanium was observed to be a requirement for the calcite carbonate formation (Figure S4) and bands in the IR-spectra assigned to a Ti-carbonate-like group (Figures 1 and 2) formed alongside BaCO_3_ (C) bands. Both observations demonstrate an interaction between the carbonate and Ti-rich intermediate phases, possibly at the interface between small domains of each phase as illustrated in Figure 7. The proposed model is further supported by the Rietveld and PDF refinements, which showed that the intermediate phases could be described by nanosized poorly crystalline BaCO_3_ (C) and BaTi_4_O_9_ with limited long-range order. TEM further demonstrated limited crystallinity and small crystallites, but diffraction from both BaCO_3_ (C) and BaTi_4_O_9_ was observed from individual (Figure 5c,e, respectively) and mixed particles (Figure 5f).

A similar model for the intermediate phases with BaCO_3_ (C) stabilized by topotaxial templating on oxygen-deficient Ti–O interface layers was suggested by Ischenko *et al*.^20^ Although the composition of the Ti-rich phase determined in this work, BaTi_4_O_9_, fits with the reported range given by Ischenko *et al*.,^19,20^ the structure of BaTi_4_O_9_ does not seem to comply with the suggested BaTiO_3_-like building blocks for the topotaxial templating model.^20^ Moreover, the refined BaCO_3_ (C) in this work has a compressed unit cell compared to the structure reported in the literature,^19,20,38^ with 0.4 % expansion of the *a*-parameter but 6.9 % compression of the *c*-parameter. This reduction in unit cell volume is due to only slight vibrations of the carbonate groups instead of full rotations. O^2–^-substitution, as suggested by Ischenko *et al*.^19,20^ to act as a second stabilizing mechanism for BaCO_3_ (C), was not investigated, but it seems likely given the deviating structure that the degree of substitution is different in this work compared to previous studies. However, since the intermediate phases are intimately mixed nanosized domains, with a certain degree of interaction between them (Figure 7), the structure could still follow the topotaxial templating model, even if the precursor chemistry and crystal structure of the intermediate phases deviate slightly. Stabilization of the calcite modification of BaCO_3_ has been reported in mixed alkaline earth carbonates^41^ or by quenching.^42^ Intermediate carbonates with the calcite structure are also reported in YBa_2_Cu_3_O_7–*x*,_^43^ Ba_1–*x*_SrTiO_3_,^11^ and Ba_1–*x*_Ca*_x_*Zr_y_Ti_1–*y*_O_3_^23^ systems. Controlling the carbonate formation was reported to be crucial for phase purity in Ba_0.85_Ca_.15_Zr_0.1_Ti_0.9_O_3_ thin films.^23^ Barium carbonate and Ti-rich intermediate phases are therefore probable intermediates during wet chemical processing of BaTiO_3_-based materials, although the exact nature of these would depend on the precursor chemistry.

### 3.3 Influence of the Heating Rate and CO_2_ on the Transformation Pathway for BaTiO_3_

Slower heating generally decreased the temperature regions for the reaction occurring during the thermal decomposition of the BaTiO_3_ precursor (Section 4.1), for certain heating rates. For fast heating (>1 °C/s), the transformation pathway is altered, and the intermediate phases are inhibited due to kinetics, giving direct BaTiO_3_ nucleation from the amorphous network. This is in line with previous results on BaTiO_3_ thin films from a similar precursor solution during fast heating (>1 °C/s).^22^ Moreover, the *in situ* IR spectra of the precursor powders (Figure S3) show that carbonate formation was less pronounced for the slow heating (0.05 °C/s), which could relate to the metastable nature of the intermediate phases and the decomposition kinetics.

Increased partial pressure of CO_2_ in the atmosphere stabilizes the intermediate phases over BaTiO_3_ nucleation (Figure 6) by drastically limiting the decomposition reaction in eq 4. For high CO_2_ partial pressures (>25 vol % CO_2_), the perovskite nucleation temperature increased more than 100 °C compared to that in synthetic air (0 vol % CO_2_). Moreover, the temperature region for the coexistence of intermediates and perovskite increased for high CO_2_ partial pressures, which means that even if the decomposition reaction in eq 4 can take place the high partial pressure of CO_2_ serves as a kinetic limitation. A second effect of a high CO_2_ partial pressure (>25 vol % CO_2_) is the formation of the thermodynamically stable BaCO_3_ (A), which once formed require temperatures above 700 °C to decompose. No secondary Ti-rich phases were observed by XRD alongside the perovskite once aragonite-type BaCO_3_ formed; hence, titanium remains as unreacted amorphous BaTi_4_O_9_. BaCO_3_ (A) formed at the same temperature as the intermediate phases; therefore, CO_2_ stabilizes a second reaction during the organic removal step described by this modified version of eq 3
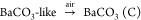
5a
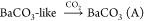
5bleading to the formation of BaCO_3_ (A) alongside the BaCO_3_ (C) phase. The formation of a broad aragonite band in the IR spectra (Figure 2a–c) before the shift toward calcite formation when the precursor powders were annealed in air (eq 5a) might be caused by locally enhanced CO_2_ partial pressure from decomposing organics. However, as the organics are further decomposed/combusted during heating, the enhanced CO_2_ partial pressure decrease before the aragonite-type BaCO_3_ can fully crystallize resulting in formation of BaCO_3_ (C). It is also likely that the apparent stability of the intermediate phases observed in the total scattering data (Figure 3) is due to locally enhanced CO_2_ partial pressure inside the capillaries during the experiments, which causes these phases to remain even during the prolonged annealing at 740 °C. Enhanced CO_2_ levels would shift the reactions along the *x*-axis in Figure 6a, which also fits with the increased reaction temperatures observed. Although it is important to note that direct comparison between the different experiments carried out in this work cannot be done due to different reaction volumes used for the different techniques. The volume used for annealing will affect the kinetics of the reactions, which is why thin films^22^ were observed to crystallize at a lower temperature than powders.^21^ However, the trends for the decomposition of the precursor and crystallization can still be discussed, independent of reaction volume.

Carbonate formation is a well-known prevailing synthesis challenge in BaTiO_3_-based materials and for Ba oxides, especially during wet chemical processing if the dissolved and atmospheric CO_2_ levels are not controlled. The structure of the carbonate forming depends on the precursor chemistry, where the BaCO_3_ (C) type is highly sensitive toward the processing conditions. However, the BaCO_3_ (C) type could be preferable over the formation of BaCO_3_ (A) due to the metastable nature of BaCO_3_ (C), which under the right processing conditions results in phase-pure BaTiO_3_ in the temperature range of 550–600 °C, as reported in this work.

## 4 Conclusions

The decomposition, pyrolysis, and crystallization reactions during synthesis of BaTiO_3_ by an aqueous-based synthesis route were characterized by *in situ* IR and synchrotron X-ray total scattering. The *in situ* analysis revealed the transformation pathway for BaTiO_3_ crystallization and the structure and composition of the intermediate metastable calcite-type BaCO_3_ and BaTi_4_O_9_ phases that formed prior to BaTiO_3_ nucleation. The crystallinity of the nanosized intermediate phases is poor as there is limited long-range order. BaTiO_3_ nucleates both directly from an amorphous network but also through the diffusion-controlled reaction of the intermediate phases. Intimate mixing of the intermediate phases and their metastable nature ensure full decomposition in controlled atmospheres. However, the stability of the intermediates over BaTiO_3_ formation is governed by the CO_2_ partial pressure, where enhanced CO_2_ levels stabilize calcite-type BaCO_3_ but also leads to the formation of the thermodynamically stable aragonite-type BaCO_3_. Therefore, control of the processing atmosphere is crucial when fabricating phase-pure BaTiO_3_ through this aqueous synthesis route. Carbonate intermediate phases by similar formation mechanisms can also be expected for BaTiO_3_-based materials and basic oxides in general.

## 5 Experimental Section

### 5.1 Synthesis

The preparation of the aqueous precursor solution has been reported previously.^21,22^ Separate Ba- and Ti-complex solutions were prepared and then mixed in stochiometric ratios to make a final BaTiO_3_ precursor solution with a concentration of 0.26 M. The Ba-solution was prepared by dissolving both EDTA (98%, Sigma-Aldrich, St. Louis, MO, USA) and citric acid (99.9%, Sigma-Aldrich, St. Louis, MO, USA) in deionized water to act as complexing agents for dissolved Ba(NO_3_)_2_ (99.9%, Sigma-Aldrich, St. Louis, MO, USA), while the Ti-solution was prepared by dissolving citric acid in deionized water followed by addition of Ti isopropoxide (97%, Sigma-Aldrich, St. Louis, MO, USA). Ammonia solution (30%, Sigma-Aldrich, St. Louis, MO, USA) was used to adjust the pH of the solutions to neutral prior to mixing.

BaTiO_3_ precursor powder was prepared by drying the precursor solution at 150 °C for 24 h in air, resulting in a sponge-like brown material, which was crushed in an agar mortar to yield the precursor power. The BaTiO_3_ precursor samples for *in situ* IR measurements were prepared by dispersing the precursor powder in deionized water and depositing droplets directly onto platinized silicon substrates (Pt/Si, SINTEF, Oslo, Norway). The droplets were flattened by draining most of the liquid of the substrate edge, leaving a wet precursor powder layer, which were dried at ambient temperature for 30–60 min. Precursor powders for *ex situ* X-ray total scattering and electron microscopy investigation of the intermediate phases were annealed at 530 °C for 1 h with a heating/cooling rate of 0.056 °C/s in air. The IR spectra of the prepared powders are included in Figure S1.

### 5.2 Characterization

Fourier-transform infrared spectra (FTIR, Vertex 80v, Bruker, Billerica, MA, USA) were recorded with a Praying Mantis Diffuse Reflection Accessory (Harrick Scientific Products Inc., Pleasantville, NY, USA) combined with the Praying Mantis High Temperature Reaction Chamber (Harrick Scientific Products Inc., Pleasantville, NY, USA). A flow of synthetic air was supplied through the reaction chamber during heating, except for one experiment, which was carried out without the dome of the reaction chamber in an ambient atmosphere and without instrument vacuum. A clean Pt/Si substrate was used as background and measured at ambient temperature under the same conditions as the samples. The spectra were recorded in reflectance mode in the range of 400–4000 cm^–1^, with a resolution of 4 cm^–1^. Each scan took ∼32 s, and the number of scans averaged depended on the heating rate; for 0.05 °C/s, 80 scans were averaged; for 0.2 °C/s, 40 scans were averaged; and for 0.5 and 1 °C/s, 20 scans were averaged. The IR spectra of the samples after heating were measured at ambient temperature in vacuum and without the dome of the reaction chamber, labeled “RT after”.

*In situ* high-temperature X-ray diffraction (HT-XRD) on the precursor powder under a controlled CO_2_ atmosphere was performed on a D8 Advance Diffractometer (Bruker, Billerica, MA, USA) with Cu Kα radiation (λ = 1.54 Å) equipped with a Vantec-1 SuperSpeed detector. The samples were prepared in a radiant heater sample holder of alumina. The partial pressure of CO_2_ in synthetic air was varied in the range of 0–100%. The sample chamber was closed and purged with the desired gas mixture for 1 h before measurements were started. The diffractograms were recorded with a step size of 0.033° and 0.5 s scan time per step during a hold step at selected temperatures, and the heating rate in between hold steps was 0.2 °C/s. Rietveld refinements of the powder XRD patterns were done with the TOPAS software (v5, Bruker, Billerica, MA, USA).

X-ray total scattering data for pair distribution function (PDF) analysis of the BaTiO_3_ precursor powders was collected on BL08W^44^ at SPring-8 (Japan) using a flat 2D panel detector and a wavelength of 0.10765 Å. The time resolution of the recorded data was 5 s. For the *ex situ* measurement, a powder preannealed at 530 °C in air was filled in a Kapton tube (OD 1.05 mm, Goodfellow, England), and for the *in situ* measurements, the precursor powders were loaded in quartz capillaries (OD 1.5 mm, CharlesSupper Company, Westborough, USA) with glass wool on each side. The capillaries had continuous air flow of 0.12 L/min and were measured in transmission. A hot air blower was used to heat the samples continuously (0.17–1 °C/s) and then held at the maximum annealing temperature for 30–70 min. The 2D images were masked and integrated with the pyFAI python package,^45^ where 5 patterns were averaged. Empty capillaries were used for background subtractions, which was done with the pdfgetx3 (v2.0.0) software using periodic structures.^46^ The *Q*-range for the samples was 16–22 Å^–1^, and the PDFs were refined with PDFGui (v1.1.2).^47^

For the transmission electron microscopy (TEM) investigation, a powder preannealed at 530 °C in air was dispersed in isopropanol in an ultrasound bath before depositing the powder particles on a holey carbon Cu-grid. TEM was performed on a JEOL JEM 2100 equipped with a LaB_6_ electron gun. Selected area diffraction patterns (SADPs) were obtained using a circular aperture covering an area in real space with a diameter of approximately 750 nm.

## Supporting Information Available

Additional infrared spectra, *in situ* total scattering data, all XRD patterns, and refined values from PDF and Rietveld refinements are listed (PDF)

## ABBREVIATIONS

as: asymmetric stretching
BaCO_3_ (A): aragonite-type BaCO_3_
BaCO_3_ (C): calcite-type BaCO_3_
BF: bright-field
BT: BaTiO_3_
CSD: chemical solution deposition
HT-XRD: high temperature X-ray diffraction
Int: intermediate
IR: infrared spectroscopy
oop: out-of-plane
PDF: pair distribution function
SADP: selected area diffraction pattern
ss: symmetric stretching
TEM: transmission electron microscopy
XRD: X-ray diffraction.
