# Supplementary material for: Structures and Role of the Intermediate Phases on the Crystallization of BaTiO3 from an Aqueous Synthesis Route
Source: ACS Omega. 2021 Mar 30;6(14):9567–76. doi: 10.1021/acsomega.1c00089 (PMC8047748; doi:10.1021/acsomega.1c00089)
Supplement: Supplementary file 1 — ao1c00089_si_001.pdf [file ao1c00089_si_001.pdf]

Supplementary Information

The structure and role of the intermediate phases on the crystallization of $BaTiO_3$ from an aqueous synthesis route

Kristine Bakken[1†], Viviann H. Pedersen[1], Anders B. Blichfeld[1†], Inger-Emma Nylund[1], Satoshi Tominaka[2], Koji Ohara[3], Tor Grande[1], and Mari-Ann Einarsrud[1*]

[1] Department of Materials Science and Engineering, NTNU Norwegian University of Science and Technology, Trondheim, Norway

[2] International Center for Materials Nanoarchitectonics, National Institute for Materials Science, Ibaraki, Japan

[3] Diffraction and Scattering Division, Center for Synchrotron Radiation Research, Japan Synchrotron Radiation Research Institute, Hyogo, Japan

Figure S1 shows the infrared (IR) spectra of the dried $BaTiO_3$ precursor solution before and after annealing at 530 °C for 1 h, showing how the intermediate carbonate phases develop during this heat treatment in the same way as during the *in situ* experiments.

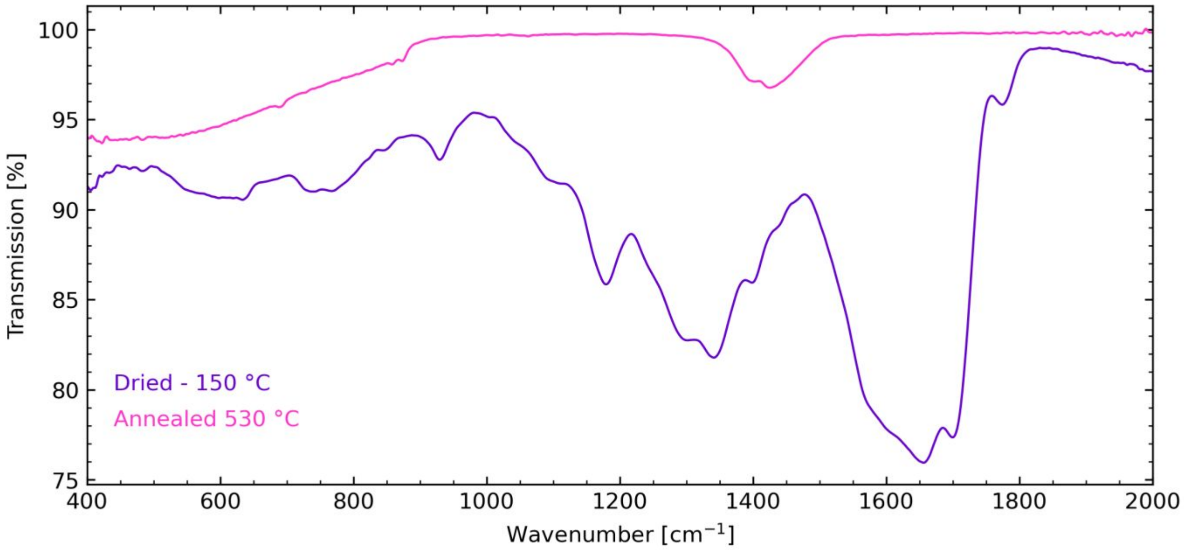

Figure S1: IR spectra of the powder made by drying the precursor solution and after annealing the dried powder at 530 °C for 1 h.

The *in situ* IR spectra of $BaTiO_3$ precursor powders with different temperature profiles are shown in Figure S2 and Figure S3. Table S1 summaries the key findings from all the *in situ* IR experiments. The carbonate oop- and as-bands appeared similar for all heating programs, although the shift in the oop-band frequency for the $BaTiO_3$ precursor with isothermal carbonate formation (Figure S3(a)-(c)) was larger. Additionally, the high frequency shoulder shifted to higher wavenumber with prolonged hold, indicating that a weaker bonding between the carbonate-like compound and the cations was developing.[1] Slow heating (Figure S3(d)-(f)) gave less pronounced carbonate bands, again accompanied by shoulders, but no frequency shift was observed for the shoulders. The shoulders were also stronger for the isothermal carbonate formation and when a low heating rate was used (Figure S3), compared to the heating program showed in Figure 2.

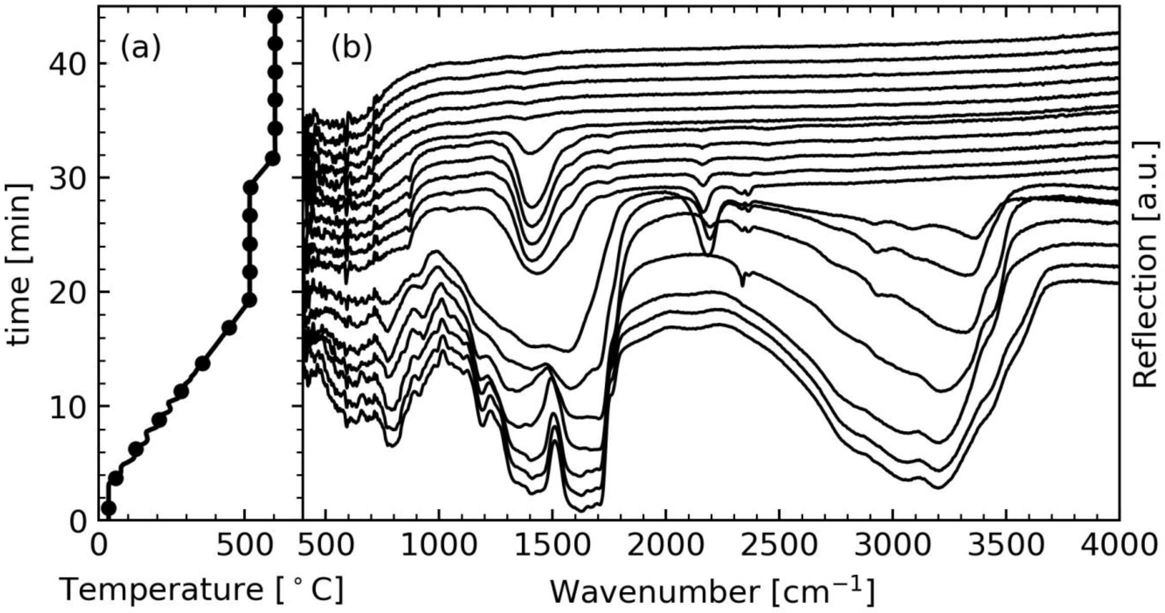

Figure S2: (a) Temperature profile and (b) corresponding *in situ* IR spectra for a $BaTiO_3$ sample.

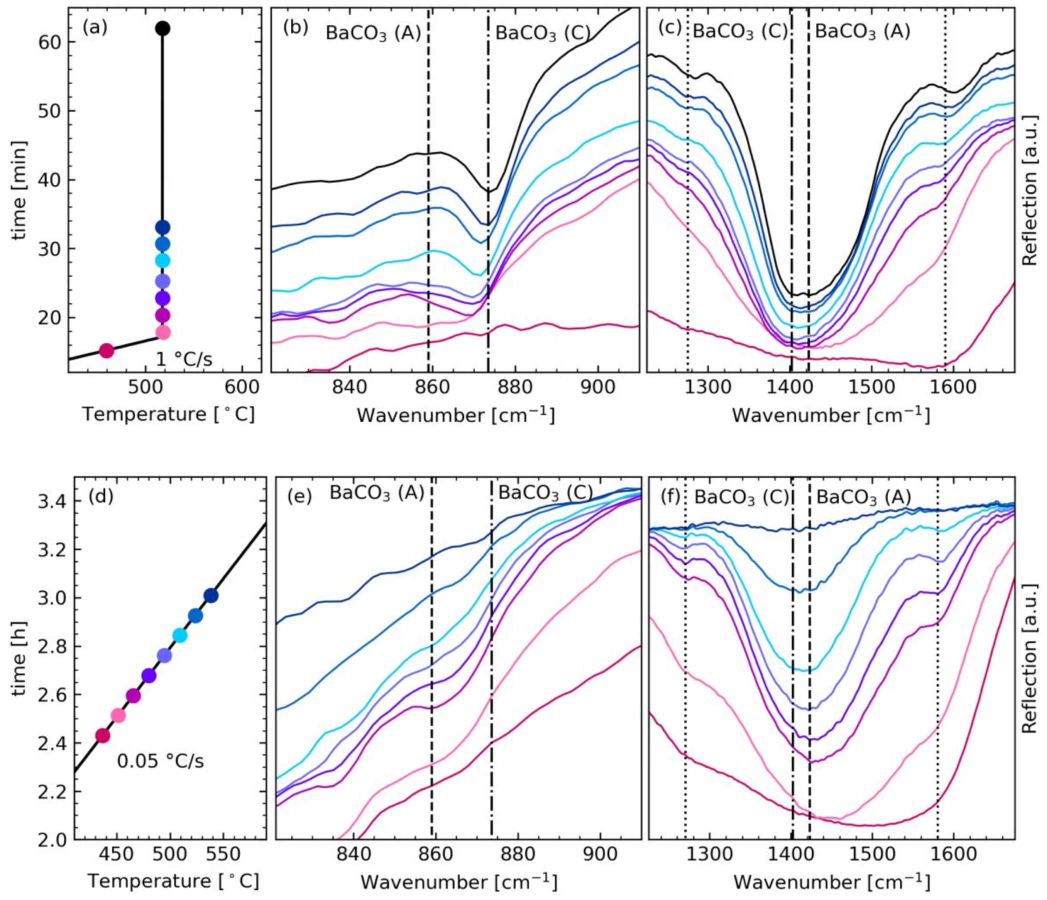

Figure S3: *In situ* IR spectra of $BaTiO_3$ precursors annealed with different temperature programs. (a) Temperature profile and IR spectra in the frequency range of (b) the out-of-plane vibrational mode and (c) the asymmetric stretching mode of $BaCO_3$, during isothermal formation of the intermediate phases. (d) Temperature profile and IR spectra in the frequency

range of (e) the out-of-plane vibrational mode and (f) the asymmetric stretching mode of $BaCO_3$ for a precursor heated continuously with a low heating rate.

Table S1. Summary of the absorption band evolution from *in situ* IR spectroscopy during annealing of $BaTiO_3$ precursor powders. All samples were heated from 20 to 610 °C.

Figure	Heating program	Carbonate oop-band [cm^{-1}]	Carbonate as-band [cm^{-1}]	Shoulders [cm^{-1}]
Figure 2(a)-(c)	0.5 °C/s w/hold	861 → 871	Broad 1430 → narrow 1402	1580
Figure 2(d)-(f)	0.2 °C/s ambient atmosphere	859 → 871	Broad 1430 → narrow 1402	none
Figure S1(a)-(c)	isothermal carbonate formation (1 °C/s)	861 → 874	Broad 1430 → narrow 1402	1280 & 1590→ 1600
Figure S1(d)-(f)	0.05 °C/s	861 → 871	Broad 1430 → narrow 1402	1270 & 1580

Figure S2 shows that if the dried single cation Ba-precursor solution is annealed at 550 °C only $BaCO_3$ (A) forms, while if the $BaTiO_3$ precursor powder is annealed at 550 °C both $BaCO_3$ (A) and $BaCO_3$ (C) is present in the powder. This demonstrates that the calcite formation is dependent on the presence of titanium during annealing of the precursor powder.

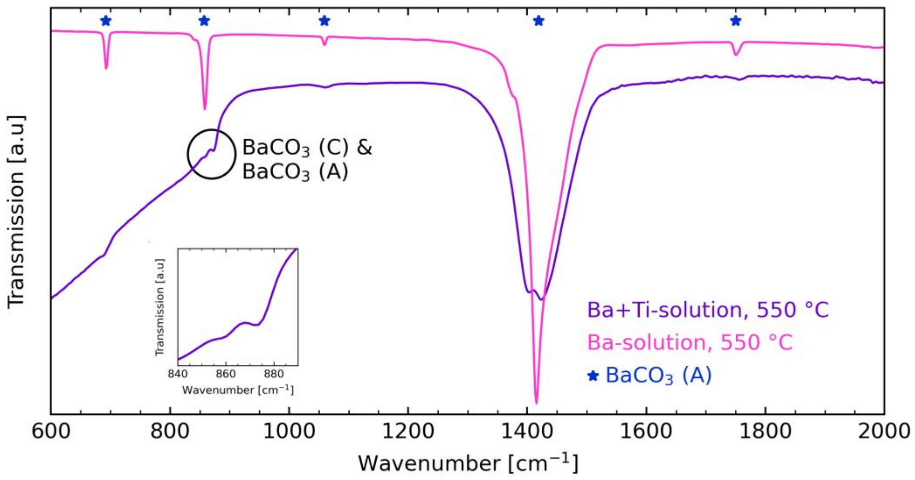

Figure S4: IR spectra of $BaTiO_3$ precursor powder and dried Ba-solution calcined at 550 °C for 2 h, showing how calcination of the Ba-solution alone results in $BaCO_3$ (A), while calcination of $BaTiO_3$ precursor powder give $BaCO_3$ (C).

In situ total scattering, converted PDFs and temperature profile for two $BaTiO_3$ samples are shown in Figure S5 and Figure S6.

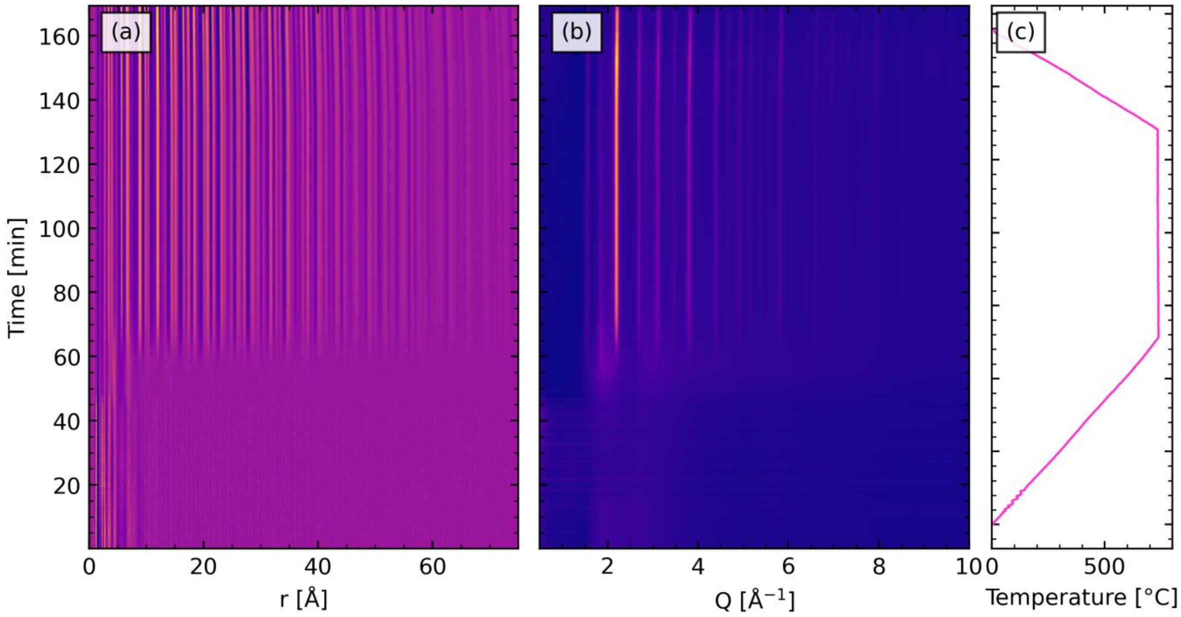

Figure S5: (a) The measured total scattering, (b) the converted PDFs and (c) temperature profile for a $BaTiO_3$ precursor powder heated with a heating rate of 0.17 °C/s to 734 °C in synthetic air.

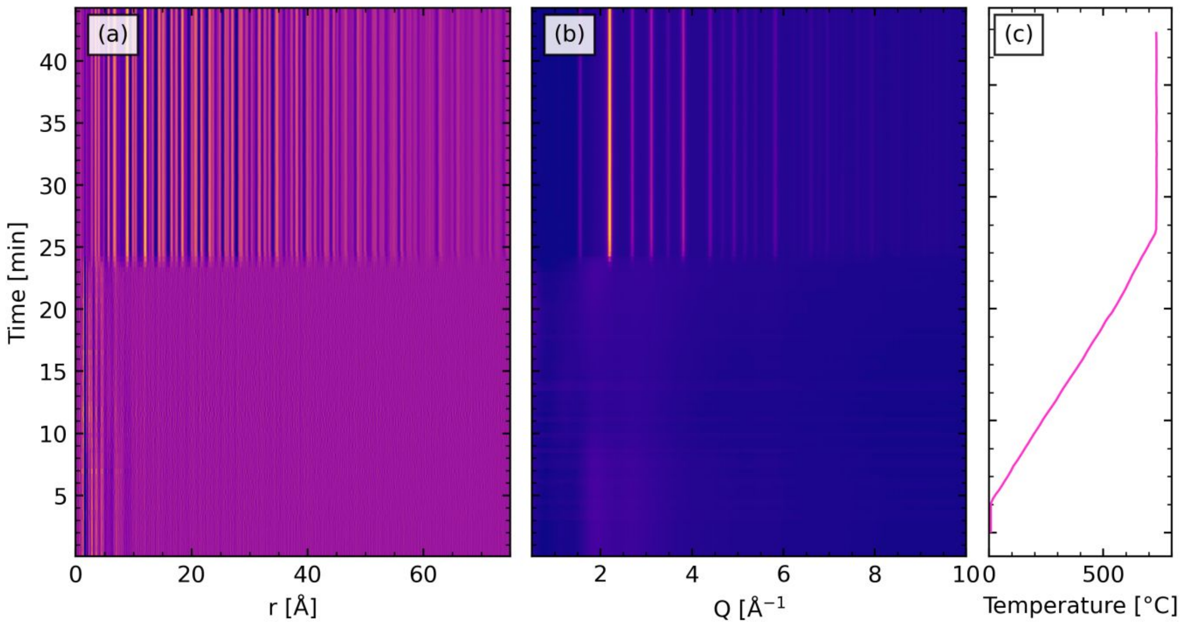

Figure S6: (a) The measured total scattering, (b) the converted PDFs and (c) temperature profile for a $BaTiO_3$ precursor powder heated with a heating rate of 1 °C/s to 727 °C in synthetic air.

The refined cell parameter from the PDF refinements of the total scattering data for the PDF showed in Figure 4 and Figure S7 are listed in Table S2. The local structure of $BaTiO_3$ was fitted to a rhombohedral structure (R3m) and the intermediate phases were fitted as $BaCO_3$ (R3m) and $BaTi_4O_9$ (Pmmn). The scale factor ratio of $BaCO_3$ to $BaTi_4O_9$ was locked to 75:25 during the refinements, for simplicity, since no significant difference between the archived lattice parameters and scale factor ratios was found when these were allowed to refine freely.

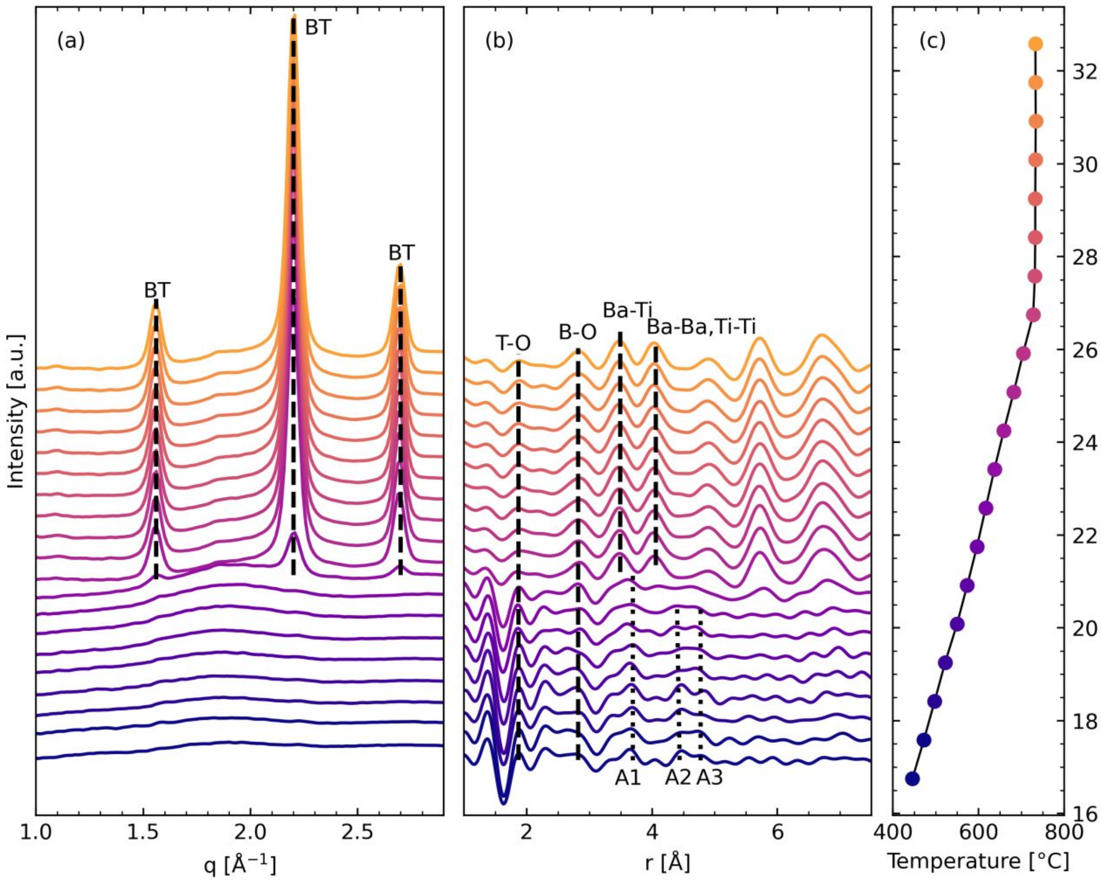

Figure S7: (a) Measured total scattering, (b) converted PDFs and (c) temperature profile for a $BaTiO_3$ precursor powder heated with a heating rate of 1 °C/s to 727 °C. The diffraction lines in (a) are indicates as “BT” for $BaTiO_3$. The atomic pair distances are indicated in (b) for the $BaTiO_3$ phase. Peak A1 corresponds to a Ba-C distance in $BaCO_3$ (A) and Ba-Ba, Ba-Ti and Ti-O distances in the $BaTi_4O_9$ phase. Peak A2 corresponds Ba-Ba and Ba-O distances in $BaCO_3$ (A). The A3 peak corresponds to a Ba-O distance in $BaCO_3$ (A) and a Ba-Ti distance in the $BaTi_4O_9$ phase.

Table S2. Results from PDF refinements of the phases that formed during annealing for $BaTiO_3$ precursor powders during the *in situ* X-ray total scattering experiments.

Powder	Phases (Group, composition)	Lattice parameters [Å]	R_{wp}
In situ heated to 740 °C with 0.17 °C/s (Figure 4(a))	$BaTiO_3$ (R3m)	a=5.7061<br>c=7.0639	33.6
	$BaCO_3$ (R3m)	a=5.2253<br>c=9.8861	
	$BaTi_4O_9$ (Pmmn)	a=13.7115<br>b=3.8276<br>c=5.9802	
In situ heated to 591 °C with 0.17 °C/s (Figure 4(b))	$BaCO_3$ (Pnma)	a=6.7679<br>b=5.30502<br>c=7.9932	71.0
	$BaTi_4O_9$ (Pmmn)	a=13.6298<br>b=3.7617<br>c=6.2882	
In situ heated to 657 °C with 0.17 °C/s (Figure 4(c))	$BaCO_3$ (R3m)	a=5.1730<br>c=9.6757	48.0
	$BaTi_4O_9$ (Pmmn)	a=13.6286<br>b=3.7621<br>c=6.2884	
In situ heated to 733 °C with 1 °C/s (Figure 4(d))	$BaTiO_3$ (R3m)	a=5.6916<br>c=7.0425	17.1
Ex situ heated to 530 °C (Figure 4(f))	$BaCO_3$ (R3m)	a=5.2445<br>c=9.7601	39.6
	$BaTi_4O_9$ (Pmmn)	a=13.5404<br>b=3.8207<br>c=6.4097	

The converted PDF and the fitted function for a $BaTiO_3$ precursor powder annealed ex situ at 530 °C for 1 h is shown in Figure S4 to illustrate that the main contribution to the PDF (and also to the diffraction pattern) originate from the $BaCO_3$ (C) phase, while the $BaTi_4O_9$ only contribute significantly for peaks below 4.5 Å.

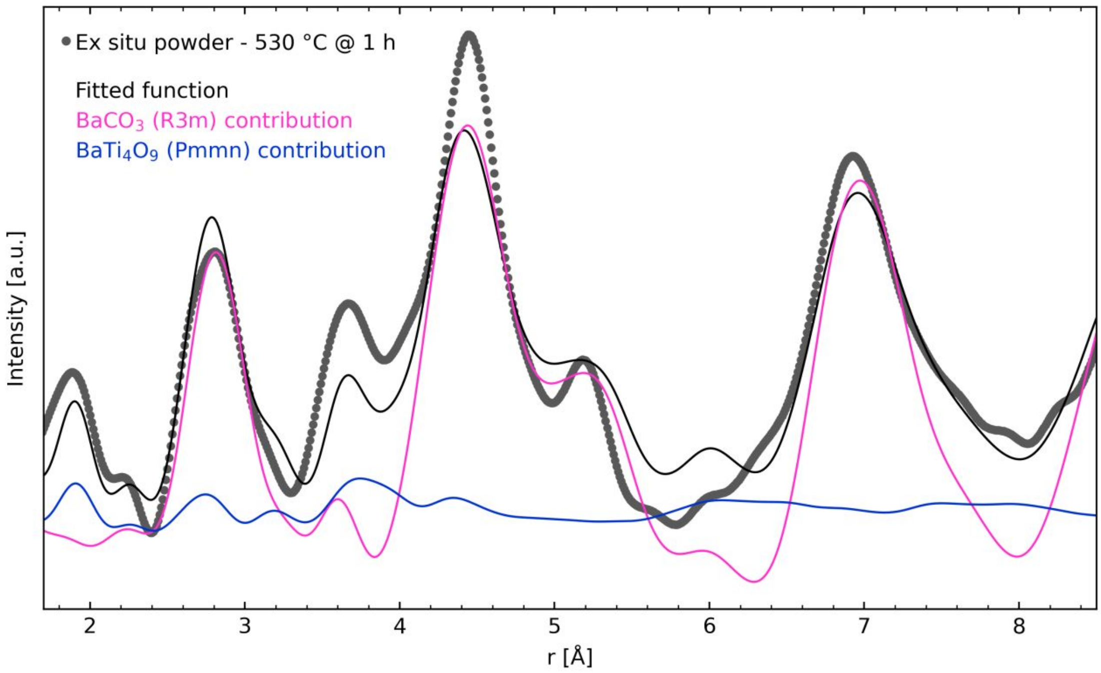

Figure S8: Fitting of the PDF of a $BaTiO_3$ precursor powder annealed *ex situ* at 530 °C for 1h. The individual contributions to the fit from the $BaCO_3$ (C) and $BaTi_4O_9$ phases are shown, where the $BaTi_4O_9$ phase only have significant contribution to the fit below 4.5 Å.

The observed d-spacings from the TEM imaging (Figure 5) of particles with the intermediate phases are listed in Table S3, and for the $BaTiO_3$ particles the calculated lattice parameter is listed for each d-spacing.

Table S3. Calculated d-spacings from the selected area diffraction patterns recorded with TEM for different particles in a $BaTiO_3$ precursor powder annealed at 530 °C for 1 h.

Particles in Figure 5 (Phases)	Calculated d-spacing [Å]								
a ($BaTiO_3$)*		2.848	2.324	1.973			2.324	1.973	
b ($BaTiO_3$)**	3.945	2.798	2.304	1.958		1.605	2.304	1.958	
c ($BaCO_3$ (C))	4.133			3.338			2.153		
d ($BaTi_4O_9$+$BaCO_3$ (A))	3.767	3.677		3.298			2.239		
e ($BaTi_4O_9$)			3.615		3.171		2.118		
f (Mixed)	4.065		3.638	3.287	3.183	2.538	2.063	2.025	1.828

*: These d-spacings corresponds to an a-parameter of 4.0282 Å (110), 4.0251 Å (111) and 3.9455 Å (200).

**: These d-spacings corresponds to an a-parameter of 3.9455 Å (100), 3.9573 Å (110), 3.9912 Å (111), 3.9168 Å (200) and 3.9325 Å (211).

The temperatures and CO_2 partial pressures used during HT-XRD measurements of $BaTiO_3$ precursor powders are shown in Figure S5(a), and the XRD patterns are displayed in Figure S5(b)-(f). The results from the Rietveld refinements of the $BaTiO_3$ precursor powders heated with 0 % CO_2 (synthetic air) and 50 % CO_2, displayed in Figure 6, are given in Table S4.

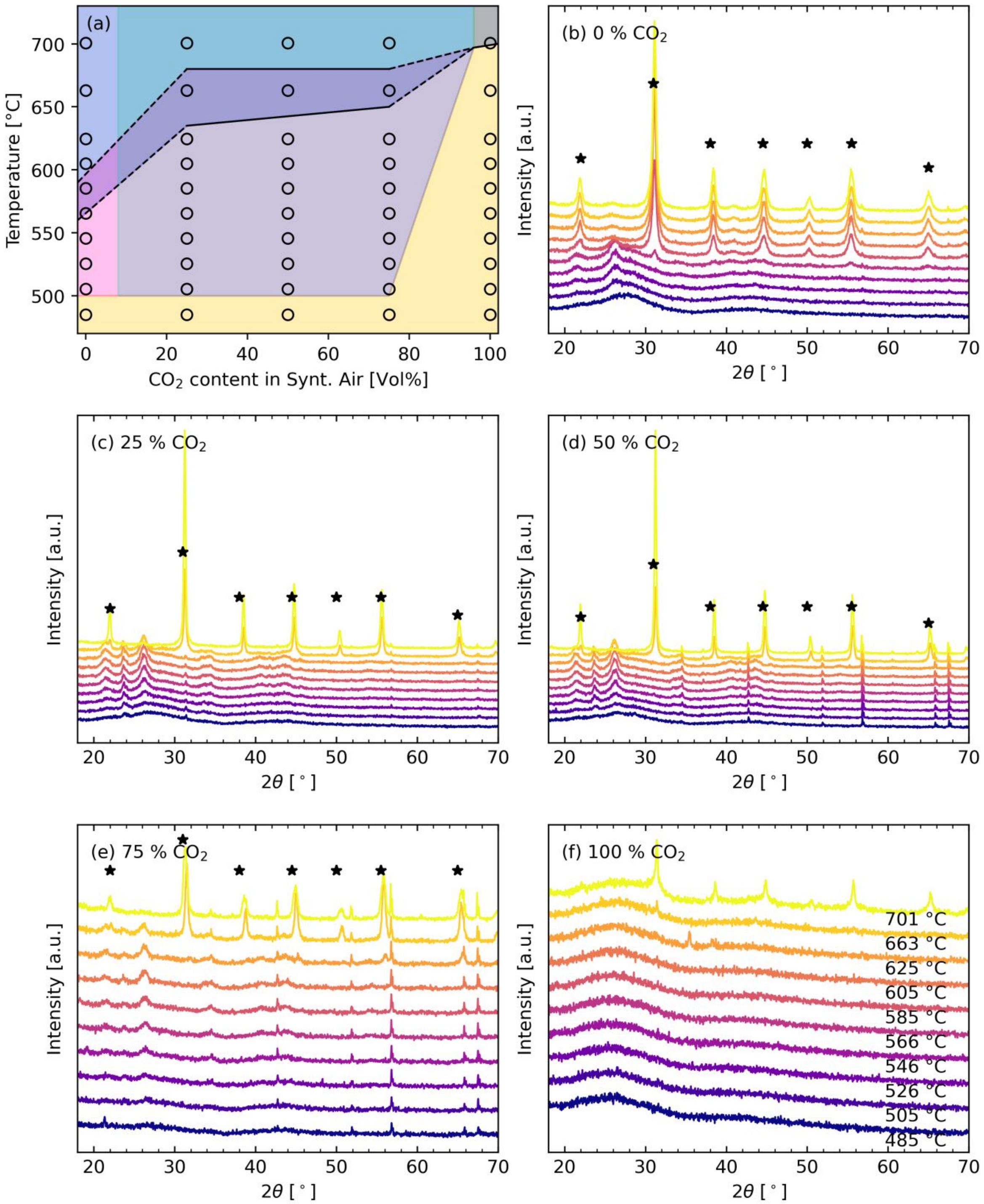

Figure S9: (a) The data points are indicated with circles during *in situ* HT-XRD measurements of $BaTiO_3$ precursor powders, where the overlaying colours illustrate the different phases presents as shown in Figure 6. The recorded XRD patterns for $BaTiO_3$ precursor annealing in (b) 0 vol% CO_2, (c) 25 vol% CO_2, (d) 50 vol% CO_2, (e) 75 vol% CO_2 and (f) 100 vol% CO_2. The diffraction peaks corresponding to cubic $BaTiO_3$ are indicated with asterisk.

Table S4. Results from Rietveld refinements of the phases that formed during annealing for $BaTiO_3$ precursor powder in variable CO_2 atmospheres.

Powder	Phases (Group, composition)	Lattice parameters [Å]	Cryst. Size [nm]	R_{wp}
546 °C in 0% CO_2 (Figure 6(b))	$BaTi_4O_9$ (Pmmn, 9.6 wt%)	a =13.7107(9), b=3.8362(2), c=6.3555(1)	28.7(4)	5.4
	$BaCO_3$ (R3m, 90.4 wt%)	a =5.2288(6), c=10.3373(2)	7.4(1)	
585 °C in 50% CO_2 (Figure 6(c))	$BaTi_4O_9$ (Pmmn, 18.9 wt%)	a =13.7466(7), b=3.8590(6), c=6.0388(5)	8.2(8)	9.6
	$BaCO_3$ (R3m, 66.0 wt%)	a =5.2325(3), c=10.3366(7)	10.5(4)	
	$BaCO_3$ (Pnma, 15.1 wt%)	a =6.6524(6), b=5.3284(7), c=8.9457(8)	32.4(2)	

PRESENT ADDRESSES

†Kristine Bakken, Materials Center Leoben Forschungs GmbH, Leoben, Austria
†Anders B. Blichfeld, Danish Technological Institute, Aarhus, Denmark

REFERENCES

1. Nakamoto, K., *Infrared and Raman spectra of inorganic and coordination compounds: Pt. B : Applications in coordination, organometallic, and bioinorganic chemistry*. 5th ed.; Wiley: New York, 1997.
